# Risk Factors for Developing Metabolic Acidosis after Radical Cystectomy and Ileal Neobladder

**DOI:** 10.1371/journal.pone.0158220

**Published:** 2016-07-06

**Authors:** Kwang Hyun Kim, Hyun Suk Yoon, Hana Yoon, Woo Sik Chung, Bong Suk Sim, Dong-Ryeol Ryu, Dong Hyeon Lee

**Affiliations:** 1 Department of Urology, Ewha Womans University School of Medicine, Seoul, Korea; 2 Department of Internal Medicine, Ewha Womans University School of Medicine, Seoul, Korea; Eberhard Karls University, GERMANY

## Abstract

**Purpose:**

To investigate the serial changes of metabolic acidosis and identify associated risk factors in patients who underwent radical cystectomy and ileal neobladder.

**Material and Methods:**

From January 2010 to August 2014, 123 patients who underwent radical cystectomy and ileal neobladder reconstruction for bladder cancer were included in this study. Metabolic acidosis was defined as a serum bicarbonate level less than 22 mEq/L and impaired renal function was defined as a GFR <50ml/min. The presence of metabolic acidosis was evaluated at 1 month, 1 year, and 2 years after surgery. Multivariate logistic regression analysis was conducted to identify risk factors associated with development of metabolic acidosis.

**Results:**

Metabolic acidosis was observed in 52%, 19.5%, and 7.3% of patients at 1 month, 1 year, and 2 years after surgery, respectively. At 1 month after surgery, impaired renal function was the only independent risk factor associated with metabolic acidosis (OR 3.87, P = 0.046). At 1 year after surgery, diabetes was the only independent risk factor associated with metabolic acidosis (OR 5.68, P = 0.002). At 2 years post-surgery, both age and diabetes were significant risk factors associated with metabolic acidosis.

**Conclusion:**

Approximately, half of patients experienced metabolic acidosis one month after ileal neobladder reconstruction. Preoperative impaired renal function was the most significant risk factor for developing metabolic acidosis in the early postoperative period. However, the incidence of metabolic acidosis decreased to less than 20% 1 year after surgery, and diabetes was an independent risk factor during this period.

## Introduction

Ileal orthotopic neobladder reconstruction is a popular method for urinary diversion after radical cystectomy, and has favorable cosmetic and quality of life outcomes [1–4]. However, neobladder reconstruction is a complicated surgery that is associated with various complications in a substantial proportion of patients. Furthermore, not all patients are ideal candidates for ileal orthotopic neobladder. Although the indications for orthotopic neobladder have been broadened, appropriate patient selection is especially important for achieving successful long term results after surgery [5]. In general, ileal orthotopic neobladder is not indicated in patients with impaired renal function due to their inability to cope with increased acid loads following absorption of urine through ileal neobladder [5, 6]. Patients with reduced renal function are vulnerable to metabolic acidosis and related complications.

Metabolic acidosis is a well-known complication in patients with intestinal reservoirs. Along with renal function, a number of different factors influence metabolic alterations after neobladder reconstruction [7]. Metabolic acidosis can have serious consequences such as negatively influencing overall bone metabolism over long term follow up [6–8]. However, most patients do not typically require treatment for metabolic acidosis for more than 3 months, and the incidence of clinically significant metabolic acidosis decreases with time after surgery [2, 9, 10].

Although several studies have evaluated metabolic changes after neobladder reconstruction, data on serial changes of acidotic status is limited [7, 11–13]. In addition, there is insufficient evidence as to whether metabolic acidosis persists or progresses in patients with impaired renal function after surgery during follow up. In this study, we investigated serial changes of metabolic acidosis in patients who underwent radical cystectomy and ileal neobladder. We also aimed to identify risk factors associated with development of metabolic acidosis during follow up.

## Materials and Methods

From January 2010 to August 2014, 149 patients underwent radical cystectomy and ileal neobladder reconstruction for bladder cancer at our institution. A retrospective review of medical record and analysis was performed after obtaining Ewha Womans University Mokdong Hospital institutional review board (2015-10-010-001). Informed consent was waived due to retrospective design and data were analyzed anonymously after removal of patient identifiers.

Of 149 patients, 26 patients who were followed up for less than 12 months or had incomplete data were excluded, resulting in a final study cohort of 123 patients. Metabolic acidosis was defined as a serum bicarbonate level less than 22 mEq/L [14]. None of the patients in our study cohort had preoperative metabolic acidosis.

The patient selection criteria for renal function was not strict, and included patients with a glomerular filtration rate (GFR) greater than 30 ml/min if they exhibited motivation and intellectual capacity. Absolute contraindications included tumor infiltration of the distal prostatic urethra in men and vagina, bladder neck or urethra invasion in women. All operations were performed using a standard open surgical approach. A Studer pouch was used for orthotopic substitution [15], and ureteroileal anastomosis was performed using the Nesbit technique. For neobladder reconstruction, an approximately 60 cm segment of ileum was isolated 20–25 cm proximal to the ileocecal valve. The ileal reservoir consisted of the distal 45 cm of the isolated segment, while the proximal 15 cm was used for the afferent limb. The urethral catheter was maintained for 10–14 days after surgery. Oral sodium bicarbonate was routinely administered after urethral catheter removal for 3 months. All patients were educated to adequately empty their neobladder and achieve a desired volume of 400–500 mL without incontinence.

Preoperative evaluation included age, sex, body mass index (BMI), comorbidities such as hypertension and diabetes, laboratory tests, and information on clinical stage. Postoperatively, patients were generally followed for the first month after surgery and every 3 months thereafter for the first two years after surgery. Routine follow up included laboratory tests as well as physical examination. GFR was assessed using a Modification of Diet in Renal Disease (MDRD) equation based on serum creatinine levels [16]. In this study, impaired renal function was defined as a GFR <50ml/min according to general guidelines [5].

The presence of metabolic acidosis was evaluated at 1 month, 1 year, and 2 years after surgery. Quantitative variables were compared using Student’s t-test and qualitative variables were compared using Chi square and Fisher’s exact tests. Serial changes in serum bicarbonate levels were assessed using repeated measures ANOVA. Multivariate logistic regression analysis was conducted to identify risk factors associated with development of metabolic acidosis at each follow-up after surgery. The Statistical Packaged for Social Science for Windows, version 18.0 (SPSS< Chicago, IL, USA) was used for all statistical analyses. A P-value <0.05 was considered significant, and all P-values were two-sided.

## Results

**Fig 1:**
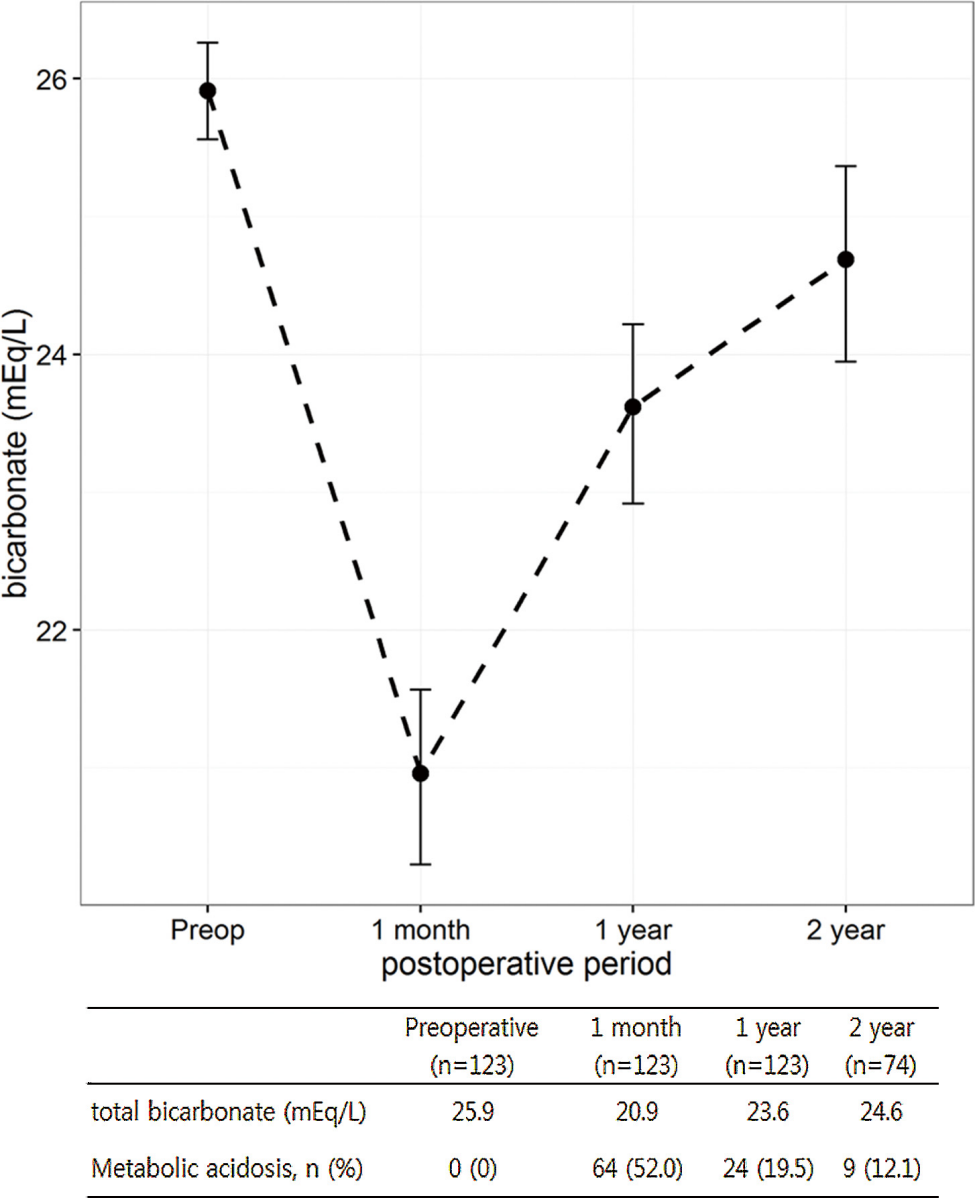
Serial changes in total bicarbonate levels and incidence of metabolic acidosis in entire cohort.

The characteristics of 123 patients included in this study are summarized in Table 1. Of 123 patients, 16 had diabetes and 14 had impaired renal function. The mean GFR was 46.2 ml/min and 77.4 ml/min in patients with impaired renal function and normal renal function, respectively. In the entire study population, the mean total bicarbonate level declined from 25.9 mEq/L to 20.9 mEq/L at 1 month after surgery, but increased thereafter to 24.6 mEq/L at 2 years after surgery. The mean total bicarbonate levels were 22.3 mEq/L, 23.5 mEq/L and 23.6 mEq/L at 3 months, 6 months and 1 year after surgery, respectively. Metabolic acidosis was observed in 52%, 19.5%, and 12.1% of patients at 1 month, 1 year and 2 years after surgery, respectively (Fig 1).

**Table 1:** Preoperative characteristics of patients.

	No. patients (%) (n = 123)
Age	
mean±SD	62.1±10.0
median (IQR)	63 (55–69)
Hypertension	43 (35.0)
Diabetes	16 (13.0)
Renal function	
impaired	14 (11.4)
normal	109 (88.6)
GFR (ml/min)	
mean±SD	73.9±17.8
median (IQR)	74.9 (63.8–82.9)
Total bicarbonate (mEq/L)	
mean±SD	25.9±1.9
median (IQR)	26 (24–27)

SD = standard deviation, IQR = interquartile range, GFR = glomerular filtration rate

**Fig 2:**
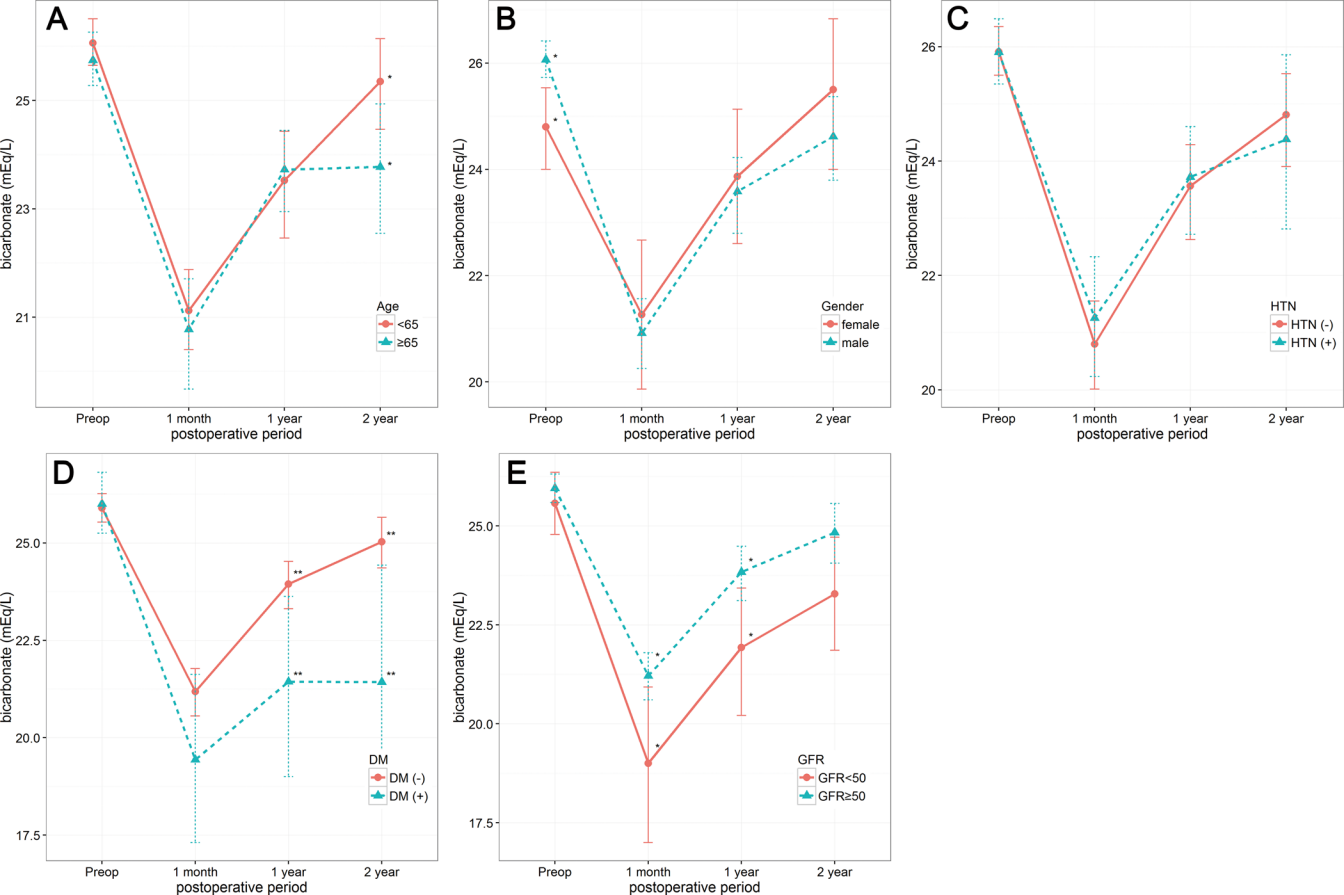
Serial changes in total bicarbonate levels according to age (A), gender (B), hypertension (C), diabetes (D) and renal function (E). *Asterisks* indicate differences between groups (*, P < 0.05; **, P < 0.01).

Serial changes in total bicarbonate according to clinical variables are shown in Fig 2. Female patients had significantly lower preoperative total bicarbonate levels, while patients older than 65 years had significantly lower total bicarbonate levels 2 years after surgery compared with patients less than 65 years of age. However, there was no difference in serial changes of total bicarbonate according to age (≥65 years vs. <65 years), gender, and hypertension. While patients with impaired renal function had significantly lower total bicarbonate at 1 month and 1 year after surgery than patients with normal renal function, total bicarbonate levels 2 year after surgery did not differ according to renal function. In patients with diabetes, total bicarbonate was significantly lower at 1 and 2 years after surgery than in patients without diabetes. Serial changes in total bicarbonate were significantly different according to presence of diabetes (p = 0.0045) and renal function (p = 0.005). We have also investigated the incidence of febrile urinary tract infection which can affect metabolic acidosis in postoperative periods. Twenty four patients had febrile urinary tract infections after surgery and 62.5% (15/24) had an episode within 1 month after surgery. However, the levels of total bicarbonate at 1 month after surgery were not significantly different between patients with and without febrile urinary tract infection within 1 month (21.0 ± 3.6 vs. 20.6 ± 2.7, P = 0.677).

When multivariate logistic regression analysis was conducted to identify risk factors for developing metabolic acidosis, impaired renal function was the only independent risk factor associated with metabolic acidosis at 1 months after surgery (OR 3.87, P = 0.046). Impaired renal function lost its significance as a risk factor at both 1 and 2 years after surgery. At 1 year after surgery, diabetes was the only independent risk factor associated with metabolic acidosis (OR 5.68, P = 0.002). At 2 years after surgery, both age and diabetes were significant risk factors associated with metabolic acidosis after controlling for gender, hypertension and preoperative renal function (Table 2).

**Table 2:** Multivariate analysis to identify risk factor for developing metabolic acidosis according to time after surgery.

	Postoperative 1 month		Postoperative 1 year		Postoperative 2 year	
	OR	P value	OR	P value	OR	P value
Age (continuous variable)	1.00 (0.96–1.04)	0.749	0.98 (0.93–1.02)	0.42	1.12 (1.01–1.26)	0.034
Gender (female vs. male)	1.27 (0.40–4.02)	0.668	1.52 (0.37–6.18)	0.552	NA	
Hypertension (yes vs. no)	0.87 (0.40–1.87)	0.722	0.70 (0.24–2.03)	0.524	2.98 (0.53–16.57)	0.212
Diabetes (yes vs. no)	1.50 (0.98–14.27)	0.466	5.68 (1.86–17.34)	0.002	7.62 (1.16–49.9)	0.034
Renal function(impaired vs. normal)	3.87 (1.02–14.65)	0.046	1.59 (0.41–6.14)	0.497	3.08 (0.20–46.1)	0.414

OR = odd ratio

## Discussion

In the present study, we evaluated the incidence of metabolic acidosis after radical cystectomy and ileal neobladder reconstruction according to serial changes in total bicarbonate levels. Metabolic acidosis is a frequent complication encountered in up to 70% of patients after ileal neobladder reconstruction, particularly during the early postoperative period [9]. Consistently, our results showed that more than half of the study patients experienced metabolic acidosis during the first month after surgery. However, the level of total bicarbonate increased thereafter, reaching preoperative levels with an overall decrease in the incidence of metabolic acidosis of 12.1% at 2 years after surgery.

Metabolic acidosis is caused by the absorption of ammonium, a proton donor, and secretion of sodium bicarbonate as a consequence of intestinal mucosa exposure to urine [12]. Various factors such as neobladder surface, incomplete emptying of the neobladder, urinary pH, and comorbidities such as impaired renal function influence the development of metabolic acidosis [7, 12, 17]. In particular, patients with impaired renal function have a reduced ability to excrete acid loads, and thus these patients have an increased risk of metabolic acidosis. Although a cut-off value for renal function has not been established, continent diversion is not generally indicated in patients with a serum creatinine >150 μmol/l or GFR <50 ml/min [5]. However, serial changes in the prevalence of metabolic acidosis have not been previously described. In this study, we noted that severe metabolic acidosis was present more frequently in patients with impaired renal function compared with those with normal renal function up to 1 year after surgery, while bicarbonate levels were not significantly different between the two groups at 2 years after surgery. These results suggest that patients with impaired renal function may be potential candidates for neobladder reconstruction if they are closely monitored and managed for metabolic acidosis during the early postoperative period. The exact mechanism underlying the decline in the incidence of metabolic acidosis is uncertain. However, structural or functional change of intestinal villi may contribute to this adaptation. In a long term follow up of neobladder reconstruction, villous atrophy was predominant, particularly in the ileal reservoir, resulting in loss of absorptive capacity [1]. Although progressive villous atrophy was also observed in the colonic reservoir, the change was less severe than the ileal reservoir [1, 18]. In addition to this structural change, chronic exposure to urine also leads to functional changes in neobladder mucosa. In an experimental study, a reduction in the number of ion transporters in the intestinal mucosa was observed as a result of chronic exposure to urine [19]. These structural and functional change can affect absorptive capacity of neobladder mucosa, resulting in decreased incidence of metabolic acidosis. Further research is necessary to better understand the underlying mechanism of this adaptation.

In the present study, we also found that patients with diabetes were at increased risk of developing metabolic acidosis with increasing time after surgery. While preoperative renal function is of importance in the early postoperative period, diabetes likely plays a more significant role with time after surgery. Interestingly, diabetes has not been previously described as a risk factor for developing metabolic acidosis in patients who have undergone neobladder reconstruction, although there are several potential explanations for this observation. First, diabetes is one of the known risk factors for renal deterioration. However, a recent study demonstrated that diabetes were associated with renal function deterioration in patients with ileal conduit diverstion but not in those with neobladder reconstruction at more than 10 years follow up [20]. In our series, although renal function decreased in patients with diabetes during the follow up period, the mean GFR was greater than 50 ml/min in patients with diabetes at 2 years after surgery. Nevertheless, patients with diabetes are at increased risk of chronic kidney disease. Specifically, diabetic nephropathy accounts for a significant component of chronic kidney disease [14]. Secondly, patients with diabetes are more prone to developing type 4 renal tubular acidosis [21]. Type 4 renal tubular acidosis is caused by impaired renal excretion or deficiency in circulating level of aldosterone, which results in defective secretion of hydrogen and potassium in the distal tubule [22]. This condition can occur even in patients with mild to moderate diabetic nephropathy, and the level of acidosis can be disproportionately severe to renal function [23]. Thirdly, there is a significant difference in the acid-base balance between patients with and without diabetes. For example, patients with diabetes have been reported to develop a less severe type of metabolic acidosis, which might be due to more efficient extrarenal generation of bicarbonate compared to non-diabetic patients [24]. However, it is possible that this finding is not applicable to patients who have an intestinal reservoir. Indeed, several experimental studies have reported that the levels of Na+-K+ ATPases, which play a key role in absorption of electrolytes, are increased in the ileal mucosa of diabetic rats [25–27]. Thus, intestinal reservoirs in patients with diabetes may behave differently in response to constant urinary exposure compared to patients without diabetes.

This study is not devoid of limitations. Due to its retrospective nature, laboratory data for metabolic profiles was not complete, and our analysis of metabolic acidosis depended on the level of serum bicarbonate. However, serum bicarbonate levels are frequently used for investigating the prevalence of metabolic acidosis, and current guidelines recommend oral supplementation with bicarbonate in chronic kidney disease patients with serum bicarbonate levels less than 22 mEq/L [14, 28]. In chronic acidosis, a low total bicarbonate level may suggest a lack of acid buffer, resulting in utilization of bone to buffer excess hydrogen ions. A recent cross-sectional study has shown that low serum bicarbonate levels were associated with lower bone mineral density even in the general population [29]. We believe the total bicarbonate level has clinical significance and could be used as a surrogate marker for metabolic acidosis in patients with ileal neobladder reconstruction, who were chronically exposed to increased acid loads.

Additional analyses including blood pH, calcium metabolism analysis, and bone mineralization would provide more information on metabolic alteration after neobladder reconstruction. Another limitation of this study was the relatively short follow-up period. Thus, it will be necessary to verify our results in a larger population with longer follow-up period.

There is a paucity of data on the long-term metabolic consequences of intestinal urinary diversions. Nevertheless, chronic metabolic acidosis can affect bone metabolism, and recent population based studies have demonstrated that patients who have undergone urinary diversion are at increased risk of fracture [30]. Although patients with impaired renal function should be monitored for metabolic acidosis more closely, our results suggest that these patients can also compensate over time if they are properly selected and meticulously managed for metabolic acidosis. In addition, patients with diabetes appear to be at a greater risk of metabolic acidosis after ileal neobladder reconstruction. Clinicians need to be aware of the possible risk of metabolic acidosis in these patients and the necessity of close monitoring.

In conclusion, approximately one half of patients experience metabolic acidosis within 1 month of undergoing radical cystectomy and ileal neobladder reconstruction. In this study, we found that preoperative impaired renal function was the most significant risk factor for developing metabolic acidosis in the early postoperative period. However, the incidence of metabolic acidosis decreased to less than 20% at 2 years, and age and diabetes were independent risk factor for developing metabolic acidosis during this period. Patients with impaired renal function may be candidates for neobladder reconstruction in certain cases, especially if they are properly managed for metabolic acidosis, particularly during the early postoperative period. With respect to long term follow up, older patients with diabetes should be carefully monitored for metabolic acidosis.
